# A closed *Candidatus* Odinarchaeum chromosome exposes Asgard archaeal viruses

**DOI:** 10.1038/s41564-022-01122-y

**Published:** 2022-06-27

**Authors:** Daniel Tamarit, Eva F. Caceres, Mart Krupovic, Reindert Nijland, Laura Eme, Nicholas P. Robinson, Thijs J. G. Ettema

**Affiliations:** 1 Laboratory of Microbiology, Wageningen University, Wageningen, the Netherlands; 2 Department of Aquatic Sciences and Assessment, Swedish University of Agricultural Sciences, Uppsala, Sweden; 3 Department of Cell and Molecular Biology, Science for Life Laboratory, Uppsala University, Uppsala, Sweden; 4 Institut Pasteur, Université Paris Cité, Centre National de la Recherche Scientifique Unité Mixte de Recherche 6047, Archaeal Virology Unit, Paris, France; 5 Marine Animal Ecology Group, Wageningen University, Wageningen, the Netherlands; 6 Laboratoire Écologie, Systématique, Évolution, Centre National de la Recherche Scientifique, Université Paris-Sud, Université Paris-Saclay, AgroParisTech, Orsay, France; 7 Division of Biomedical and Life Sciences, Faculty of Health and Medicine, Lancaster University, Lancaster, UK

**Keywords:** Metagenomics, Metagenomics, Archaeal genomics

## Abstract

Asgard archaea have recently been identified as the closest archaeal relatives of eukaryotes. Their ecology, and particularly their virome, remain enigmatic. We reassembled and closed the chromosome of *Candidatus* Odinarchaeum yellowstonii LCB_4, through long-range PCR, revealing CRISPR spacers targeting viral contigs. We found related viruses in the genomes of diverse prokaryotes from geothermal environments, including other Asgard archaea. These viruses open research avenues into the ecology and evolution of Asgard archaea.

## Abstract (web summary)

The closed chromosome of an Asgard archaeon, *Candidatus* Odinarchaeum yellowstonii LCB_4, revealed CRISPR spacers, which were used to identify archaeal viruses.

## Main

Asgard archaea are a diverse group of microorganisms that comprise the closest relatives of eukaryotes^1–6^. Their genomes were first explored seven years ago^7^ and much of their physiology and cell biology is unknown. While over 200 Asgard archaeal draft genomes are available, most are represented by highly fragmented and incomplete metagenome-assembled genomes (MAGs), which has precluded obtaining insights into their mobile genetic elements (mobilome). Given the central role of Asgard archaea in eukaryogenesis models, access to their complete genomes and information about their interactions with viruses are highly relevant. In the present article, we report the closed genome of a thermophilic Asgard archaeon and the consequent discovery of complete bona fide Asgard archaeal viruses.

**Extended Data Fig. 1:**
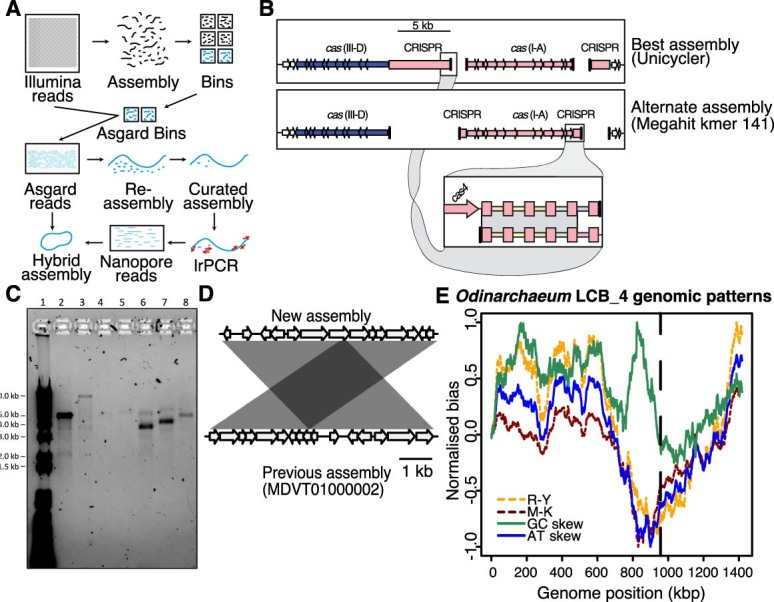
Obtaining a closed *Ca*. Odinarchaeum LCB_4 chromosome. **(a**) Summary methodology for the reassembly, refinement and closing of the *Ca*. Odinarchaeum LCB_4 genome. (**b**) Schematic of the assembly status before long-range PCR (lrPCR), indicating the presence of gaps and the agreement between two separate assemblies, which guided primer design. (**c**) Purified lrPCR products; lane 1: Invitrogen 1 kb Plus DNA ladder (Thermo Fisher Scientific Inc), 2: Positive control ca. 5 kbp rRNA gene cluster; 3: Positive control ca. 10 kbp ribosomal protein gene cluster; 4-5: first gap closing, at distances of ca. 5 and 5.5 kbp; 6-8: second gap closing, at distances of ca. 4, 4.5 and 5 kbp. Bands of the same sizes were observed 3 times following different cycling parameters, with the clearest visualization shown in this gel. (**d**) Comparison between previous assembly and new assembly for Huginn virus, indicating circularity. Similarity lines represent two single BlastN hits with up to 1 mismatches. (**e**) Genomic patterns of the *Ca*. Odinarchaeum LCB_4 indicating a potential origin of replication at position 959350.

**Extended Data Fig. 2:**
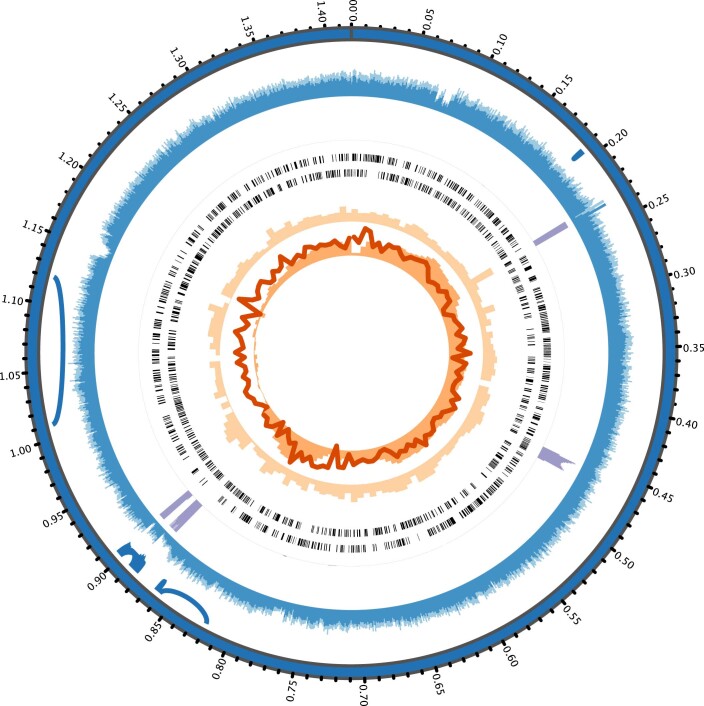
Genome map of *Ca*. Odinarchaeum LCB_4. From inside out: (1) GC skew (line) and cumulative GC skew (histogram); (2) GC content; (3) Crick strand genes; (4) Watson strand genes; (5) Nanopore reads coverage capped at 1500X; (6) Illumina read coverage (light: proper pairs, NM < 3) capped at 50X; (7) repeats; (8) chromosome contig.

To obtain a complete Asgard archaeal genome, we reassembled the genome of strain LCB_4, originally classified as the founding member of the Odinarchaeota, a 1.46 mega base pair (Mbp) assembly distributed in 9 contigs^1^. A promising reassembly yielded a 1.41 Mbp contig, a 13 kilo base pair (kbp) contig containing CRISPR-associated (Cas) genes, and multiple short contigs harbouring mobile elements or repeat signatures (Extended Data Fig. 1 and Supplementary Table 1). After contig boundary inspection, we postulated that the first two contigs represented the entire chromosome DNA sequence since these were flanked by similar CRISPR arrays that extended for several kbp. We successfully amplified these gaps using long-range PCR, sequenced the resulting amplicons with Nanopore sequencing and performed a hybrid assembly, finally generating a single 1.418 Mbp circular contig (Extended Data Fig. 2). Given the high quality of this genome, we suggest recognizing this strain as *Candidatus* Odinarchaeum yellowstonii LCB_4 (hereafter LCB_4), in reference to Yellowstone National Park, the location of the hot spring where it was sampled (Supplementary Text 1).

**Fig. 1:**
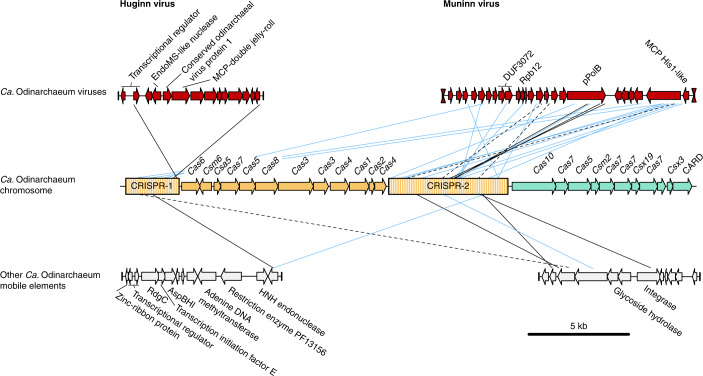
*Ca*. Odinarchaeum LCB_4 CRISPR–Cas system and mobile elements. CRISPR–Cas systems in the *Ca*. Odinarchaeum LCB_4 chromosome (centre) coloured according to their type classification (orange: I-A; aquamarine: III-D). Full contigs representing mobile elements are shown at the corners, with the vertical lines representing contig boundaries. Viral terminal inverted repeats are represented by hourglass symbols. Connecting lines represent significant full-coverage spacer hits against mobile element targets, shown in black if detected by BlastN (no mismatches: full; one mismatch: dashed) and blue if detected by SpacePHARER and not overlapping with those in black. Source data

**Extended Data Fig. 3:**
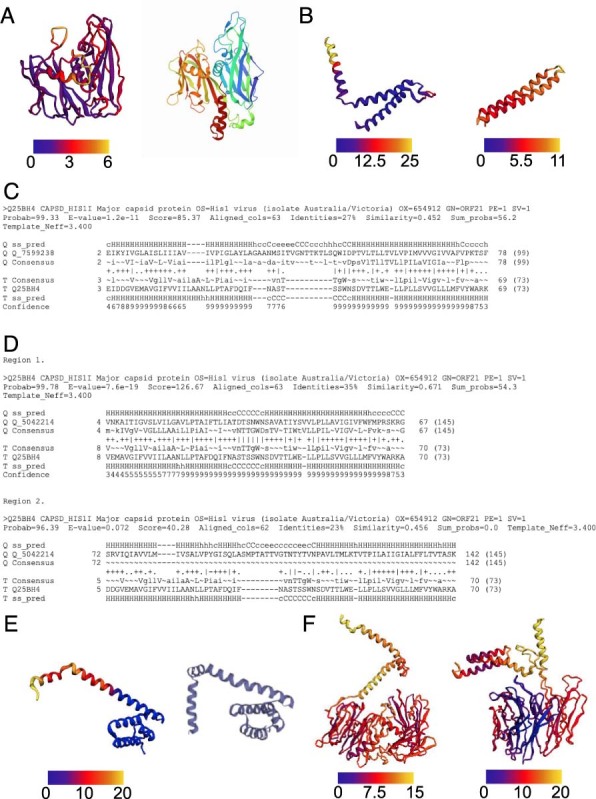
Predicted structure of selected proteins. Comparisons between the structures of (**a**) DJR-MCPs (left: Huginn virus: OLS18934.1; right: Sulfolobus turreted icosahedral virus 1: 3J31); (**b**) His1-like MCPs (left: Muninn virus: OLS18630.1; right: His1 virus: YP_529533.1); (**e**) SIRV2-like MCPs (left: Jordarchaeia QZMA23B3: QZMA23B3_25900; right: Sulfolobus islandicus rod-shaped virus 2 (SIRV-2): 3J9X) and (f) transmembrane proteins (left: Muninn virus: OLS18631.1; right: *Ca*. Odinarchaeum LCB_4 virus: OLS16720). All structures predicted with RoseTTAFold are color-coded according to their error estimate (Å). (**c,d**) Given the high error estimates for the predicted structures of His1-like MCPs, we append HHsearch results for (C) OLS18630.1 (Muninn virus) and (D) OLS18934.1 (*Ca*. Odinarchaeum LCB_4 MAG), the latter of which shows a tandem duplication (Regions 1 and 2) of the His1-like MCP. H(h), α-helix; E(e), β-strand; C(c), coil.

The LCB_4 genome contains a complex CRISPR–Cas gene system (Fig. 1), including neighbouring type I-A and type III-D Cas gene clusters, separated by a 6.1-kbp-long type I-A CRISPR array and further followed by another 2.7-kbp-long type I-A CRISPR array, with a total of 142 CRISPR 35–42 bp spacers across both arrays. Nine of these spacers targeted (with 100% identity and query coverage) 4 putative mobile element contigs obtained in the same assembly that were not part of the closed chromosome (Fig. 1 and Supplementary Tables 1 and 2), all of which had *Ca*. Odinarchaeum predicted as the host by WIsH^8^. In addition, we identified multiple poorer matches from spacers using SpacePHARER^9^ (Fig. 1), possibly representing interactions with diverged relatives of these elements. Two of these contigs contained genes encoding common mobile element proteins, such as restriction endonucleases and integrases, but did not contain any obvious viral signature genes (Supplementary Table 3). A third contig represented a complete, circular viral genome (Extended Data Fig. 1d) encoding transcriptional regulators, an endonuclease and a double jelly-roll major capsid protein (MCP), typical of tailless icosahedral viruses (Fig. 1, Extended Data Fig. 3a and Supplementary Table 3). This specific protein was previously found in a study of the double jelly-roll MCP family and tentatively named an ‘Odin group’ of sequences given this protein’s origin in the same metagenome as *Ca*. Odinarchaeum LCB_4 (ref. ^10^). The complete recovery of LCB_4’s CRISPR arrays allowed us to confirm that this circular contig indeed represents a virus associated with *Ca*. Odinarchaeum (Supplementary Table 4), for which we suggest the name ‘Huginn virus’, in reference to one of two ravens of Odin, Huginn (‘thought’).

Furthermore, 3 spacers yielded full-coverage, identical matches (and a further 3 spacers with 1 mismatch) against a 12.7-kbp-long contig recovered by the *Ca*. Odinarchaeum LCB_4 reassembly (Fig. 1). All three hits targeted an open reading frame encoding a protein-primed family B DNA Polymerase (pPolB), a gene frequently observed in archaeal viruses. Further inspection of this contig revealed genes encoding a zinc-ribbon protein and a His1-like family MCP (Extended Data Fig. 3b–d and Supplementary Table 3), conserved in spindle-shaped viruses^11^. This contig had a coverage over 3 times higher than that of the chromosome, suggestive of viral DNA replication, and was flanked by approximately 80-nucleotide-long terminal inverted repeats, a typical signature of viruses with linear double-stranded DNA genomes replicated by pPolBs^12^. Thus, this contig represents a complete Asgard archaeal viral genome for which we suggest the name ‘Muninn virus’ (Supplementary Table 4), in relation to the second raven of Odin, Muninn (‘memory’).

**Fig. 2:**
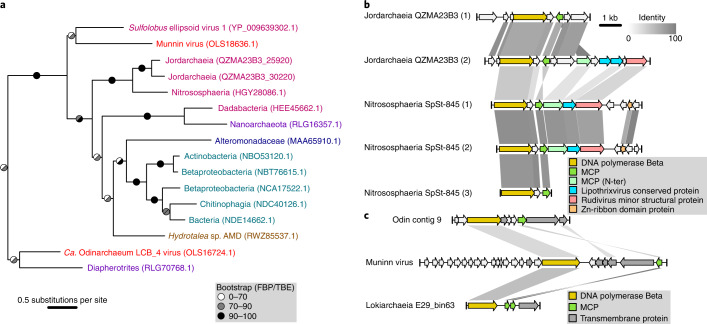
Discovery of additional Asgard archaeal mobile elements. **a**, Phylogeny of pPolB obtained with IQTree2 under the Q.pfam+C60+R4+F+PMSF model. Colours: *Ca*. Odinarchaeum LCB_4 MAG (red); sequences obtained from hot springs (pink); hydrothermal vents (purple); marine water (dark blue); Chatahoochee river (USA) (light blue); mine drainage (brown). Branch support values are FBP (left) and TBE (right). The tree presented is a clade of the full tree shown in Supplementary Fig. 1. **b**,**c**, Comparison between the viral contigs of Jordarchaeia QZMA23B3 and Nitrososphaeria SpSt-845 (**b**) and of Muninn virus and viral contigs in the bins of *Ca*. Odinarchaeum LCB_4 and Lokiarchaeia E29_bin63 (**c**). Gene map similarity lines represent reciprocal BlastP hits with an E-value lower than 1 × 10^−5^ and percentage identity as shown in the upper-right legend.

**Extended Data Fig. 4:**
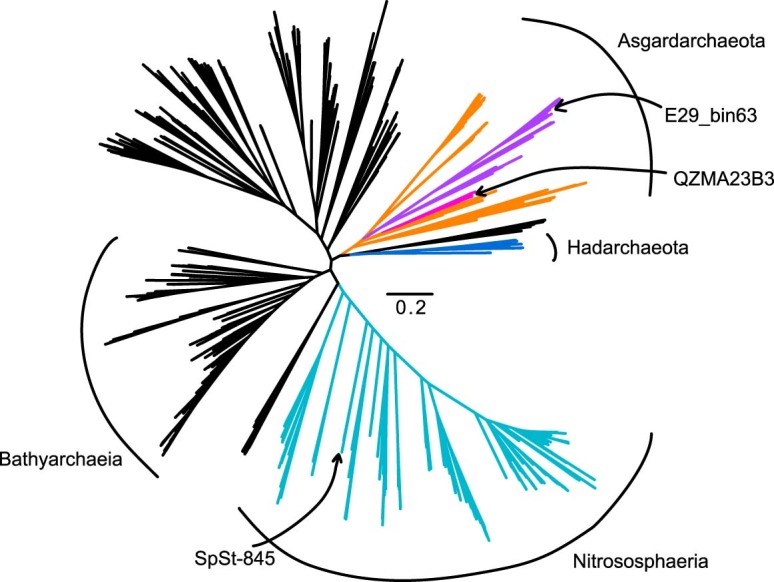
Taxonomic placement of archaeal MAGs. Phylogenomic tree obtained with FastTree including three archaeal MAGs (arrows) containing viral contigs and GTDB Archaea representatives for the phyla Hadarchaeota, Asgard archaea and Thermoproteota. Branch colors within Asgard archaea (orange) represent Jordarchaeia (pink) and Lokiarchaeia (purple). All placements are supported with branch support values of 1.0. Full tree can be found in data repository (see Data Availability statement).

We further queried the pPolB sequence from the Muninn virus genome through phylogenetic analysis, finding that it is closely related to a homologue in *Sulfolobus* ellipsoid virus 1 (SEV1)^13^ (Fig. 2a and Supplementary Fig. 1), recently isolated from a Costa Rican hot spring. No other genes were shared between Muninn virus and SEV1, which is indicative of recent horizontal transfer of *polB* in at least one of these viruses. Interestingly, other close homologues included multiple sequences that were likewise obtained from hot springs or hydrothermal vents (Fig. 2a). Two of these hits were part of an Asgard archaeal MAG (QZMA23B3), and a third pPolB homologue (HGY28086.1) belonged to a MAG (SpSt-845) originally classified as Bathyarchaeota. A phylogenomic analysis indicated that QZMA23B3 belonged to the recently described Asgard archaeal class Jordarchaeia^6^ and that SpSt-845 in fact belonged to the Nitrososphaeria (Extended Data Fig. 4). Closer inspection of the Nitrososphaerial MAG revealed 2 additional pPolB sequences from the same MAG that were highly similar (>80% identity) to HGY28086.1. The five pPolB homologues were encoded in contigs containing *Sulfolobus islandicus* rod-shaped virus 2 (SIRV2) family MCP genes (Fig. 2b, Extended Data Fig. 3e and Supplementary Table 3), exclusive to archaeal filamentous viruses with linear double-stranded DNA genomes and classified into the realm *Adnaviria*^14^. Both the Jordarchaeia and Nitrososphaeria contigs displayed high conservation in synteny and protein sequences, indicating high contig completeness and recent diversification (Fig. 2b). Notably, none of the known archaeal viruses with SIRV2 family MCPs encodes its own pPolB, suggesting that the group identified herein represents a previously undescribed archaeal virus family. However, while we detected CRISPR arrays in the MAGs where these viral contigs were identified, we could not find accurate spacer matches (query coverage >90%, identity >90%) to these viral sequences; therefore, the identity of the hosts of these thermophilic viruses is unclear.

The pPolB phylogeny further suggests that a clade of viral sequences found in MAGs from mesophiles evolved from a likely thermophile-infecting ancestor. While none of the mentioned mobile elements share other proteins in common with Muninn virus, a more distant relative of the Muninn virus pPolB sequence was found in a contig from the same LCB_4 assembly. Like Muninn virus, this sequence encoded a His1-like MCP and a gene encoding a transmembrane protein of unknown function (Fig. 2c). These two genes surrounded another gene encoding a relatively long protein (>550 amino acid residues) with multiple transmembrane helices and complex predicted structures (Extended Data Fig. 3f), with no detectable similarity but possibly related functions. We further queried the His1-like MCPs for detectable homologues, finding only a small Lokiarchaeial contig encoding two His1-like MCPs that are 83–85% identical to the Muninn virus MCP, plus a phylogenetically distant pPolB (Supplementary Fig. 1) and a protein of unknown function (Fig. 2c).

The CRISPR–Cas system of *Ca*. Odinarchaeum yellowstonii LCB_4 is likely its primary antiviral defence system. We could find no homologues for DISARM^15^ or other recently discovered antiviral systems^16,17^ in its genome. The retention of many CRISPR spacers against these mobile elements is significant and indicates coevolutionary dynamics with viruses from multiple families.

Two additional studies identifying Asgard archaeal viruses accompany ours. Rambo et al.^18^ described viruses belonging to the Caudoviricetes class, while Medvedeva et al.^19^ described three groups of viruses, of which two, skuldviruses and wyrdviruses, are distantly related to the Huginn and Muninn viruses, respectively, and are associated with Lokiarchaeal hosts. The sets of viruses found by these three studies thus complement each other.

Our findings highlight the benefits of improving the quality of Asgard archaeal genomes. The discovery of viruses of thermophilic Asgard archaea expands our limited knowledge of the Asgard archaeal mobilome^18–20^ and promises exciting advances in the study of the ecology, physiology and evolution of the closest archaeal relatives of eukaryotes.

## Methods

### *Ca.* Odinarchaeon LCB_4 genome reassembly

To reassemble the *Ca.* Odinarchaeon LCB_4 genome (Supplementary Fig. 1a), its corresponding Illumina reads^21^ (BioSample SAMN04386028) were mapped against Asgard archaeal MAGs using Minimap2 (ref. ^22^) v.2.2.17. Mapped reads were extracted and assembled with Unicycler^23^ v.0.4.4. Unicycler tested *k*-mer lengths ranging from 27 to 127; the latter was chosen to perform an assembly with default parameters. This assembly obtained a 1.406 Mbp contig, which was not predicted as circular despite both of its contig boundaries ending in type I-A CRISPR arrays (Supplementary Fig. 1b). Additional short (<13 kbp) contigs were not considered part of the main chromosome because they represented mobile elements (with signatures such as differing coverage, circularity, CRISPR spacer hits and/or presence of typical mobile element genes), ribosomal RNA genes from other organisms or CRISPR arrays (the latter two were expected due to the conservation of rRNA gene sequences and CRISPR repeats). After removing these contigs, only 1 additional contig of 10.6 kbp containing type I-A Cas genes remained. Given that the 1.406 Mbp contig ended in type I-A CRISPR arrays, we hypothesized that these two contigs could represent the entire circular chromosome of *Ca*. Odinarchaeum LCB_4. In parallel, we assembled the Illumina reads with MEGAHIT^24^ v.1.1.3 (--*k*-min 57 --*k*-max 147 --*k*-step 12). While highly fractionated, this assembly found an alternative solution for the sequences involved in the contig borders of the previous assembly. Particularly, inspecting the assembly performed with *k*-mer 141 we observed that the type I-A Cas genes were surrounded by 2 separate CRISPR arrays. Moreover, four consecutive spacers in the innermost side of one of the CRISPR arrays in this assembly were identical to the outermost spacers of the CRISPR array present at the border of the 1.406 Mbp contig in the Unicycler assembly (Supplementary Fig. 1b). These results suggested a specific disposition for the two aforementioned contigs.

### Long-range PCR and Nanopore sequencing

Four regions were selected for long-range PCR: two contig gaps, corresponding to CRISPR arrays, and two control regions spanning approximately 5 kbp of the rRNA operon and approximately 10 kbp of a ribosomal protein gene cluster (Supplementary Table 2). Primers were designed using OligoEvaluator (http://www.oligoevaluator.com/OligoCalcServlet) (Sigma-Aldrich) and synthesized by Integrated DNA Technologies. Multiple displacement amplification-amplified environmental DNA isolated from the Lower Culex Basin at Yellowstone National Park^21^ was then amplified with Herculase polymerase (Agilent Technologies). Amplification of control and gap regions was then performed following the parameters shown in Supplementary Tables 5 and 6. Products were separated on a 0.8% agarose gel in 1× Tris-Borate-EDTA buffer stained with SYBR-Gold and purified using a QIAGEN Spin purification kit according to the manufacturer’s instructions. Purified PCR fragments were pooled and used to construct a library with the SQK-LSK109 ligation kit. Sequencing was performed on an Oxford Nanopore MinION Mk1C sequencer using an R9.4.1 flow cell. Raw sequence data were basecalled using Guppy v.4.2.2. Reads were separated in 2 bins at 3–9 kbp (subsampled to 30×) and 9–12 kb and processed to obtain consensus sequences using Decona^25^ v.0.1.2 (-c 0.85 -w 6 -i -n 25 -M -r). Both control regions, comprising the rRNA and ribosomal protein operons, were 100% identical to the corresponding nucleotide sequences of the published assembly.

### Hybrid assembly

Reads were filtered using NanoFilt v.2.6.0 with the options "-q 10 -l 1000". We used these filtered Nanopore reads and the mapped Illumina reads to perform a hybrid assembly with Unicycler v.0.4.4, which resolved both the main chromosomal contig and a viral contig (Huginn virus) as circular (Supplementary Fig. 1d,e). Read mapping was performed using Bowtie 2 (ref. ^26^) v.2.3.5.1 for Illumina reads and minimap2 (ref. ^22^) v.2.17.r941 for Nanopore reads. A local cumulative GC skew minimum (Supplementary Fig. 1f), together with low R–Y (purine minus pyrimidine), M-K (amino minus keto) and cumulative AT skew values, was selected as a potential replication origin; the circular contig was permutated to set this position as nucleotide +1.

### Annotation

CRISPR arrays were detected and classified using CRISPRDetect^27^ v.2.4 and Cas genes were detected and classified through CRISPRcasIdentifier^28^ v.1.1.0. Spacer similarity searches were assessed against IMG/VR^29^ v.3 (release 5.1) and against all available databases on the CRISPRTarget^30^ webserver on 26 January 2022. Local spacer searches were performed using BlastN^31^ v.2.10.0+ (-task blastn-short) against the *Ca*. Odinarchaeum assembly, its source metagenome and the nucleotide National Center for Biotechnology Information (NCBI) database. SpacePHARER^9^ v5-c2e680a was used to search against the *Ca.* Odinarchaeum assembly and the 2018 GenBank phage and eukaryotic virus databases facilitated by the software, using as control sequences the eukaryotic virus database (with reversed sequences when using this database as target). WIsH^8^ v.1.1 was used to predict host sequences of mobile element contigs, using *Ca*. Odinarchaeum and all archaeal representative genome sequences from the Genome Taxonomy Database (GTDB)^32^ release 202. VirSorter2 (ref. ^33^) v2.2.3 was run with default parameters on the mobile element contigs. Proteins were classified into Clusters of Orthologous Groups (COG) families^34^ based on five best local BlastP^31^ v.2.10.0+ hits to the same COG; domain annotation was performed through InterProScan^35^ v.5.48-83.0. Mobile element protein annotation was performed using HHsearch^36^ v.3.3.0 against Pfam^37^ v.33.1, Protein Data Bank^38^ (16 November 2020), SCOPe^39^ (01 March 2017), CDD^40^ v.3.18 and UniProt^41^ vir70 (10 August 2020) viral protein sequence databases. Synteny plots were performed with genoPlotR^42^ v.0.8.11. Structural predictions were performed with RoseTTAFold^43^ through the Robetta portal.

### Phylogenetics

Reference pPolB sequences were obtained from Kim et al.^44^ and used for Psi-blast^45^ v.2.10.0+ against the NR v5 (as of 10 February 2021) database. Sequences with over 70% similarity were removed with CD-Hit^46^ v.4.7. The remaining sequences were aligned with Mafft-linsi^47^ v.7.450; columns with over 50% gaps were removed using trimAl^48^ v.1.4.rev22. Additionally, sequences with over 50% gaps in the trimmed alignment were removed. Maximum-likelihood trees were reconstructed using IQ-TREE^49^ v.2.0-rc1 and its implementation of ModelFinder^50^ with all combinations of the empirical models LG, JTT, WAG and Q.pfam with the site class mixtures (none, C20, C40, C60), rate heterogeneity (none, G4 and R4) and frequency (none, F) parameters. Using the obtained tree as a guide, a posterior mean site frequency (PMSF)^51^ approximation of the selected model (Q.pfam + C60 + R4 + F) was used to reconstruct a tree with 100 non-parametric bootstrap pseudo-replicates, which was then interpreted both as the standard Felsenstein bootstrap proportion (FBP) and as transfer bootstrap expectation (TBE)^52^. Double jelly-roll and His1-like MCPs were separately searched with Psiblast using the alignments of query sequences and references from Yutin et al.^10^ or hits from individual BlastP searches. No further Asgard archaeal double jelly-roll MCPs and only two Lokiarchaeial His1-like MCPs were found.

To assess the taxonomy of selected MAGs with contigs encoding homologues to the Munnin and Huginn viral proteins, all Thermoproteota, Hadarchaeota and Asgard archaea GTDB^53^ representative sequences (as of 1 February 2022) were retrieved and supplemented with Asgard archaeal sequences from the Hermod^54^, Sif^4^, Wukong^5^ and Jord^6^ groups. Together with the query sequences, GToTree^55^ v.1.5.45 was then used to reconstruct a tree with the parameters -H Archaea -D -G 0.2.

### Reporting summary

Further information on research design is available in the Nature Research Reporting Summary linked to this article.

## Supplementary information

Supplementary Information Supplementary information and Fig. 1.

Reporting Summary

Supplementary Tables 1–6 Table 1. Analysis of contigs longer than 5 kbp obtained from an assembly of Illumina reads using Unicycler v.0.4.4. The blue rows correspond to contigs that were merged using long-range PCR, Nanopore sequencing and hybrid assembly to close the Odinarchaeum chromosome. The orange rows represent bona fide extrachromosomal mobile elements. Table 2. CRISPR spacer analysis. CRISPR spacer information was obtained from CRISPRDetect and is shown separately for the two CRISPR arrays (Fig. 1). Additional columns include similarity searches performed with Spacepharer (orange) and BlastN (blue). Table 3. Annotation of mobile elements. Orange cells represent contigs containing CRISPR spacer targets from *Ca.* Odinarchaeum, while grey cells represent contigs with homologous pPolB. Yellow cells represent annotation of key viral proteins. Table 4. Minimal information for uncultivated viruses (MIUViGs) associated to new viruses. Table 5. Primers used for long-range PCR. Table 6. Long-range PCR cycling parameters.

## Data Availability

Raw Nanopore amplicon reads and the complete *Ca*. Odinarchaeum LCB_4 assembly are available at the NCBI under BioProject no. PRJNA319486. Additional data and supporting alignments and trees can be found at 10.6084/m9.figshare.19131413 (ref. ^56^). Source data are provided with this paper.

## Source data

Source Data Fig. 1 Non-inverted image corresponding to the gel in Extended Data Fig. 1c.

## Code availability

No custom code was required for the analyses in this manuscript.

## References

[1] Zaremba-Niedzwiedzka K (2017). Asgard archaea illuminate the origin of eukaryotic cellular complexity. Nature.

[2] Williams TA, Cox CJ, Foster PG, Szöllősi GJ, Embley TM (2020). Phylogenomics provides robust support for a two-domains tree of life. Nat. Ecol. Evol..

[3] Eme L, Spang A, Lombard J, Stairs CW, Ettema TJG (2017). Archaea and the origin of eukaryotes. Nat. Rev. Microbiol..

[4] Farag IF, Zhao R, Biddle JF (2021). “*Sifarchaeota*,” a novel Asgard phylum from Costa Rican sediment capable of polysaccharide degradation and anaerobic methylotrophy. Appl. Environ. Microbiol..

[5] Liu Y (2021). Expanded diversity of Asgard archaea and their relationships with eukaryotes. Nature.

[6] Sun JE (2021). Recoding of stop codons expands the metabolic potential of two novel Asgardarchaeota lineages. ISME Commun..

[7] Spang A (2015). Complex archaea that bridge the gap between prokaryotes and eukaryotes. Nature.

[8] Galiez C, Siebert M, Enault F, Vincent J, Söding J (2017). WIsH: who is the host? Predicting prokaryotic hosts from metagenomic phage contigs. Bioinformatics.

[9] Zhang R (2021). SpacePHARER: sensitive identification of phages from CRISPR spacers in prokaryotic hosts. Bioinformatics.

[10] Yutin N, Bäckström D, Ettema TJG, Krupovic M, Koonin EV (2018). Vast diversity of prokaryotic virus genomes encoding double jelly-roll major capsid proteins uncovered by genomic and metagenomic sequence analysis. Virol. J..

[11] Krupovic M, Quemin ERJ, Bamford DH, Forterre P, Prangishvili D (2014). Unification of the globally distributed spindle-shaped viruses of the Archaea. J. Virol..

[12] Krupovic M, Cvirkaite-Krupovic V, Iranzo J, Prangishvili D, Koonin EV (2018). Viruses of archaea: structural, functional, environmental and evolutionary genomics. Virus Res..

[13] Wang H (2018). Novel *Sulfolobus* virus with an exceptional capsid architecture. J. Virol..

[14] Krupovic M (2021). *Adnaviria*: a new realm for archaeal filamentous viruses with linear A-form double-stranded DNA genomes. J. Virol..

[15] Ofir G (2018). DISARM is a widespread bacterial defence system with broad anti-phage activities. Nat. Microbiol..

[16] Bernheim A (2021). Prokaryotic viperins produce diverse antiviral molecules. Nature.

[17] Doron S (2018). Systematic discovery of antiphage defense systems in the microbial pangenome. Science.

[18] Rambo I, De Anda V, Langwig M, Baker B (2022). Genomes of six viruses that infect Asgard archaea from deep-sea sediments. Nat. Microbiol..

[19] Medvedeva S (2022). Three families of Asgard archaeal viruses identified in metagenome-assembled genomes. Nat. Microbiol..

[20] Wu F (2022). Unique mobile elements and scalable gene flow at the prokaryote–eukaryote boundary revealed by circularized Asgard archaea genomes. Nat. Microbiol..

[21] Baker BJ (2016). Genomic inference of the metabolism of cosmopolitan subsurface Archaea, Hadesarchaea. Nat. Microbiol..

[22] Li H (2018). Minimap2: pairwise alignment for nucleotide sequences. Bioinformatics.

[23] Wick RR, Judd LM, Gorrie CL, Holt KE (2017). Unicycler: resolving bacterial genome assemblies from short and long sequencing reads. PLoS Comput. Biol..

[24] Li D (2016). MEGAHIT v1.0: a fast and scalable metagenome assembler driven by advanced methodologies and community practices. Methods.

[25] Doorenspleet, K. et al. High resolution species detection: accurate long read eDNA metabarcoding of North Sea fish using Oxford Nanopore sequencing. Preprint at *bioRxiv*10.1101/2021.11.26.470087 (2021).

[26] Langmead B, Salzberg SL (2012). Fast gapped-read alignment with Bowtie 2. Nat. Methods.

[27] Biswas A, Staals RHJ, Morales SE, Fineran PC, Brown CM (2016). CRISPRDetect: a flexible algorithm to define CRISPR arrays. BMC Genomics.

[28] Padilha VA, Alkhnbashi OS, Shah SA, de Carvalho A, Backofen R (2020). CRISPRcasIdentifier: machine learning for accurate identification and classification of CRISPR–Cas systems. Gigascience.

[29] Paez-Espino D (2017). IMG/VR: a database of cultured and uncultured DNA viruses and retroviruses. Nucleic Acids Res..

[30] Biswas A, Gagnon JN, Brouns SJJ, Fineran PC, Brown CM (2013). CRISPRTarget: bioinformatic prediction and analysis of crRNA targets. RNA Biol..

[31] Camacho C (2009). BLAST+: architecture and applications. BMC Bioinformatics.

[32] Parks DH (2022). GTDB: an ongoing census of bacterial and archaeal diversity through a phylogenetically consistent, rank normalized and complete genome-based taxonomy. Nucleic Acids Res..

[33] Guo J (2021). VirSorter2: a multi-classifier, expert-guided approach to detect diverse DNA and RNA viruses. Microbiome.

[34] Galperin MY, Makarova KS, Wolf YI, Koonin EV (2015). Expanded microbial genome coverage and improved protein family annotation in the COG database. Nucleic Acids Res..

[35] Jones P (2014). InterProScan 5: genome-scale protein function classification. Bioinformatics.

[36] Steinegger M (2019). HH-suite3 for fast remote homology detection and deep protein annotation. BMC Bioinformatics.

[37] Mistry J (2021). Pfam: the protein families database in 2021. Nucleic Acids Res..

[38] Burley SK (2021). RCSB Protein Data Bank: powerful new tools for exploring 3D structures of biological macromolecules for basic and applied research and education in fundamental biology, biomedicine, biotechnology, bioengineering and energy sciences. Nucleic Acids Res..

[39] Chandonia J-M, Fox NK, Brenner SE (2019). SCOPe: classification of large macromolecular structures in the structural classification of proteins-extended database. Nucleic Acids Res..

[40] Lu S (2020). CDD/SPARCLE: the conserved domain database in 2020. Nucleic Acids Res..

[41] Bateman A (2021). UniProt: the universal protein knowledgebase in 2021. Nucleic Acids Res..

[42] Guy L, Kultima JR, Andersson SG (2010). genoPlotR: comparative gene and genome visualization in R. Bioinformatics.

[43] Baek M (2021). Accurate prediction of protein structures and interactions using a three-track neural network. Science.

[44] Kim J-G (2019). Spindle-shaped viruses infect marine ammonia-oxidizing thaumarchaea. Proc. Natl Acad. Sci. USA.

[45] Schäffer AA (2001). Improving the accuracy of PSI-BLAST protein database searches with composition-based statistics and other refinements. Nucleic Acids Res..

[46] Fu L, Niu B, Zhu Z, Wu S, Li W (2012). CD-HIT: accelerated for clustering the next-generation sequencing data. Bioinformatics.

[47] Katoh K, Standley DM (2013). MAFFT multiple sequence alignment software version 7: improvements in performance and usability. Mol. Biol. Evol..

[48] Capella-Gutiérrez S, Silla-Martínez JM, Gabaldón T (2009). trimAl: a tool for automated alignment trimming in large-scale phylogenetic analyses. Bioinformatics.

[49] Minh BQ (2020). IQ-TREE 2: new models and efficient methods for phylogenetic inference in the genomic era. Mol. Biol. Evol..

[50] Kalyaanamoorthy S, Minh BQ, Wong TKF, von Haeseler A, Jermiin LS (2017). ModelFinder: fast model selection for accurate phylogenetic estimates. Nat. Methods.

[51] Wang H-C, Minh BQ, Susko E, Roger AJ (2018). Modeling site heterogeneity with posterior mean site frequency profiles accelerates accurate phylogenomic estimation. Syst. Biol..

[52] Lemoine F (2018). Renewing Felsenstein’s phylogenetic bootstrap in the era of big data. Nature.

[53] Parks DH (2020). A complete domain-to-species taxonomy for Bacteria and Archaea. Nat. Biotechnol..

[54] Zhang J-W (2021). Newly discovered Asgard archaea Hermodarchaeota potentially degrade alkanes and aromatics via alkyl/benzyl-succinate synthase and benzoyl-CoA pathway. ISME J..

[55] Lee MD (2019). GToTree: a user-friendly workflow for phylogenomics. Bioinformatics.

[56] Tamarit, D. et al. A closed *Candidatus* Odinarchaeum chromosome exposes Asgard archaeal viruses. Dataset. *figshare*10.6084/m9.figshare.19131413 (2022). 10.1038/s41564-022-01122-y PMC9246712 35760836

