# Supplementary material for: A closed Candidatus Odinarchaeum chromosome exposes Asgard archaeal viruses
Source: Nat Microbiol. 2022 Jun 27;7(7):948–52. doi: 10.1038/s41564-022-01122-y (PMC9246712; doi:10.1038/s41564-022-01122-y)

nature portfolio

https://doi.org/10.1038/s41564-022-01122-y

Supplementary information

A closed *Candidatus* Odinarchaeum chromosome exposes Asgard archaeal viruses

In the format provided by the authors and unedited

Brief communication – *Nature Microbiology*

A closed *Candidatus* Odinarchaeum chromosome exposes Asgard archaeal viruses

Daniel Tamarit[1,2]*, Eva F. Caceres[3], Mart Krupovic[4], Reindert Nijland[5], Laura Eme[6], Nicholas P. Robinson[7], Thijs J. G. Ettema[1]*

SUPPLEMENTARY MATERIAL

Supplementary Text S1. Proposal of type genome and higher taxonomic ranks.

Additional Supplementary Figure S1. pPolB phylogeny. Midpoint-rooted full tree corresponding to Fig. 2A. Taxon labels shown in Fig 2A are colored with the same pattern, and their corresponding branches are colored red. The pPolB of Lokiarchaeia E29_bin63 (Fig. 2C) is marked in orange. Support values are transfer bootstrap expectation (left) and Felsenstein bootstrap proportions (right). Full tree and sequence files can be found in data repository (see Data Availability statement).

SUPPLEMENTARY TEXT S1

Proposal of type genome and higher taxonomic ranks

***Candidatus* Odinarchaeum yellowstonii**

Candidatus Odinarchaeum yellowstonii (yel.low.sto'nii. N.L. neut. adj. *yellowstonii*: of or pertaining to Yellowstone National Park (USA), location of the hot spring where it was sampled. This uncultured lineage is represented by the strain "LCB_4", with a complete genome (NCBI BioSample SAMN04924820, Genbank accession number CP091871), recovered from hot spring sediments. Its genome is based on a 1,418,364 bp-long chromosome, and contains one copy of each of 23S, 16S and 5S rRNA genes and 13 tRNAs.

***Candidatus* Odinarchaeum gen. nov.**

Candidatus Odinarchaeum (O.din.ar.chae'um. N.L. neut. n. *archaeum* archaeon; N.L. neut. n.*Odinarchaeum* an archaeon named after Odin, the god of the heavens, battle, poetry and death, and the chief of all gods in Norse mythology). Inferred to be a thermophilic lineage with streamlined genomes. Type species: *Candidatus* Odinarchaeum yellowstonii, based on the genome for strain LCB_4, with GenBank assembly accession CP091871.

***Candidatus* Odinarchaeaceae fam. nov**.

Ca. Odinarchaeaceae (O.din.ar.chae.ace'ae. N.L. neut. n. *Odinarchaeum*, Candidatus generic name; -*aceae*, ending to designate a family; N.L. fem. pl. n. *Odinarchaeaceae*, the *Odinarchaeum* family). Type genus is *Candidatus Odinarchaeum* with the type species *Candidatus Odinarchaeum* yellowstonii, based on the genome for strain LCB_4, with GenBank assembly accession CP091871.

***Candidatus* Odinarchaeales ord. nov**. Odinarchaeales (O.din.ar.chae.a'les. N.L. neut. n. *Odinarchaeum*, Candidatus generic name; -*ales*, ending to designate an order; N.L. fem. pl. n. *Odinarchaeales*, the *Odinarchaeum* order). Type family is *Candidatus Odinarchaeaceae.* The description is the same as for *Candidatus* Odinarchaeaceae fam. nov.

***Candidatus* Odinarchaeia class. nov**. Odinarchaeia (O.din.ar.chae'i.a. N.L. neut. n. *Odinarchaeum*, Candidatus generic name; -*ia*, ending to designate a class; N.L. neut. pl. n. *Odinarchaeia*, the *Odinarchaeum* class). Type order is *Candidatus* Odinarchaeales. The description is the same as for *Candidatus* Odinarchaeales ord. nov.

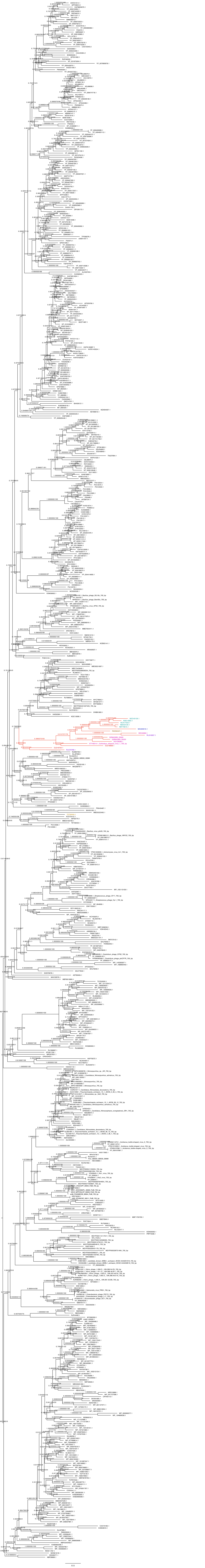

Supplement: Supplementary file 1 — Supplementary information and Fig. 1. [file 41564_2022_1122_MOESM1_ESM.pdf]
