# Supplementary figures and images for: A closed Candidatus Odinarchaeum chromosome exposes Asgard archaeal viruses
Source: Nat Microbiol. 2022 Jun 27;7(7):948–52. doi: 10.1038/s41564-022-01122-y (PMC9246712; doi:10.1038/s41564-022-01122-y)

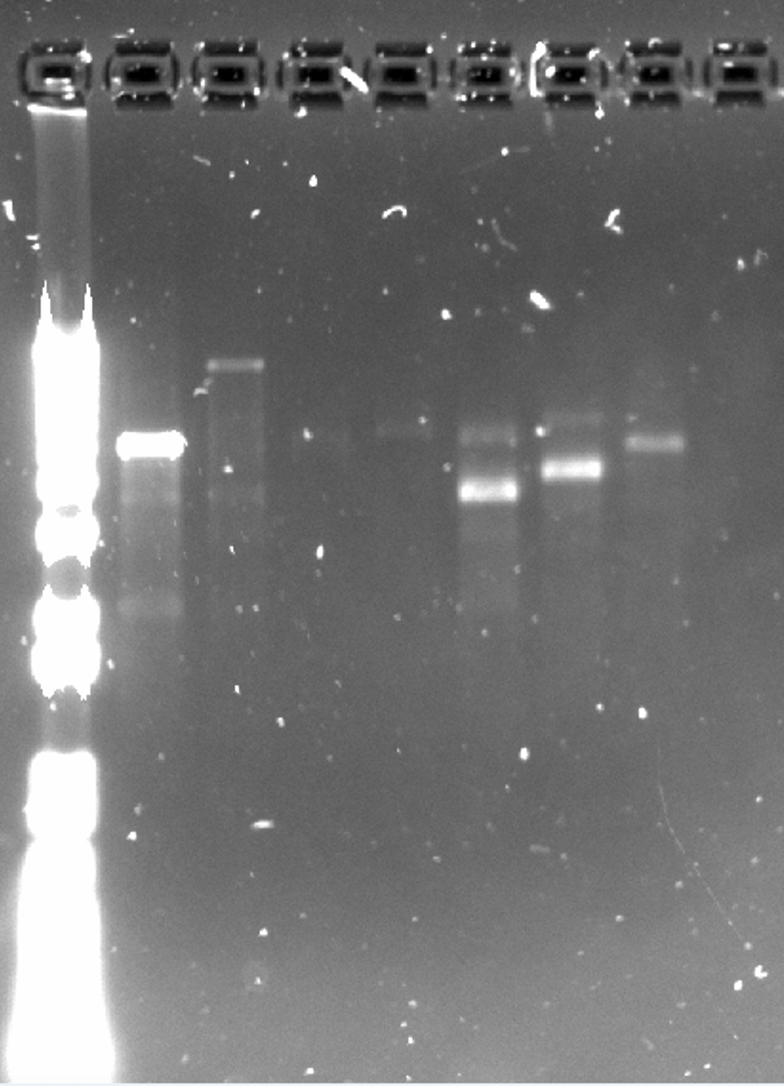

Supplement: Source Data Fig. 1 — Non-inverted image corresponding to the gel in Extended Data Fig. 1c. [file 41564_2022_1122_MOESM4_ESM.tiff]
